# Nanobubble Formation by Flow Regime Switching Using a Tesla Valve

**DOI:** 10.1021/acsomega.4c10246

**Published:** 2025-04-14

**Authors:** George Joseph, Bincy Binny, Andre R. Venter

**Affiliations:** Department of Chemistry, Western Michigan University, Kalamazoo, Michigan 49008-5413, United States

## Abstract

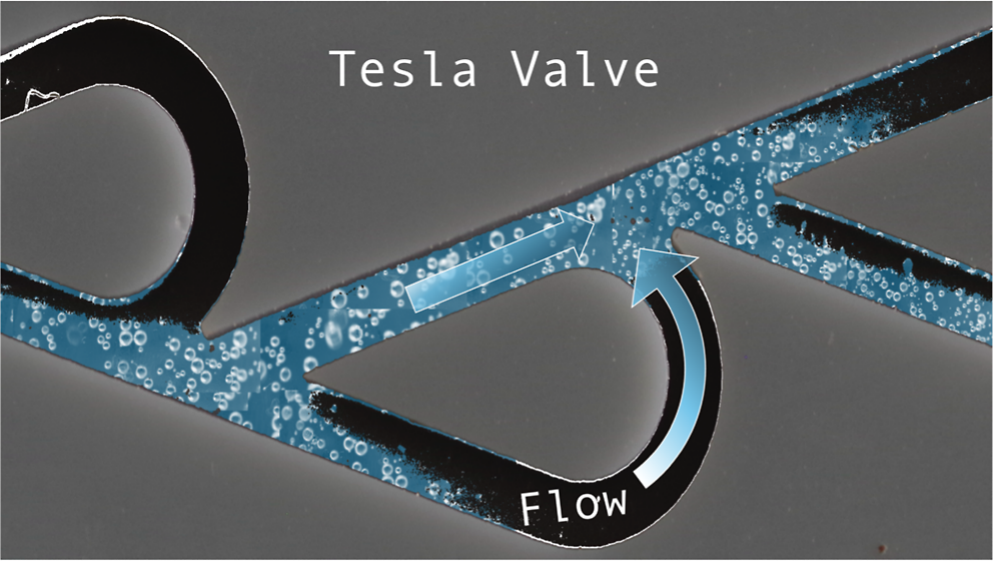

Nanobubbles (NBs) are very small gas cavities in solution, and when their sizes reach diameters around 200 nm, they exhibit special qualities with widespread application. We introduce a novel, cost-effective method for the generation of nanobubbles by flow regime cycling through a Tesla valve, a valvular condiut without moving parts. We compare the performance of Tesla valve flow regime cycling with other previously reported methods for laboratory-scale NB generation. NBs were created from CO_2_ or N_2_ and by flow regime swiching using the Tesla valve, ultrasonication, and pressure cycling. The comparison includes bubble diameter, bubble concentration, and zeta potential under individually optimized conditions. The average bubble diameter generated by the Tesla valve from CO_2_ was measured at 110 nm by NTA, which is similar to sonication but small compared to the bubbles produced by the pressure cycling method (140 nm). Additionally, the average concentration of bubbles created by the Tesla valve was 3.8 × 10^8^ bubbles/mL, more than sonication at 3.0 × 10^8^ bubbles/mL but fewer than pressure cycling at 2.6 × 10^9^ bubbles/mL. The surface charge was recorded at −33 mV, just below sonication at −36 mV but larger than pressure cycling at −21 mV. The results indicate that flow cycling through a Tesla valve generates NBs in the 100–200 nm range, which compares favorably to the alternative laboratory-scale methods while promising low energy consumption and easy scalability for future industrial applications.

## 1 Introduction

Bubbles below 1 μm exhibit special properties when their diameters approach 200 nm and are referred to as nanobubbles (NBs). Unlike microbubbles, frequently characterized as bubbles with a diameter between 10 and 50 μm, NBs do not coalesce to burst at liquid surfaces but can remain stable in solution for extended periods of time.^1^ These concavities exhibit characteristics such as low buoyancy, negative surface charges, the formation of free radicals, enhanced water mobility, high internal pressure, enormous surface to volume ratio, and high gas dissolution rate. A thermodynamic study at room temperature and pressure indicates that an aqueous bulk bubble with a diameter smaller than 180 nm does not begin to dissolve spontaneously.^2^ Even so, NBs hold the promise of overcoming the solubility barrier by providing a significant source of dissolved gases, for example CO_2_ or air, in solution.^3^

In general, the zeta potential measurement of micro- and nanobubbles are negative, but the values change with different types of gases,^4,5^ solvents,^6^ and solvent additives such as surfactants and electrolytes.^7^ In pure water, the zeta potential for CO_2_ NBs in water ranges between −21 and −36 mV. Other gases such as air (−17 to −20 mV), nitrogen (−29 to −35 mV), oxygen (−34 to −45 mV), and xenon (−11 to −22 mV) also have negative zeta potentials.^4^ It has been suggested that the negative zeta potential of NBs leads to the formation of an electrical double layer, contributing to their long life times.^8,9^ Satpute and Earthman proposed that bulk nanobubble stability in water is a likely consequence of balancing the surface tension of a shrinking microbubble with the electrostatic repulsion of hydroxyl ions, which initially adsorb onto the precursor microbubble surface before shrinking.^9^ Other models and their limitations are discussed in several review papers.^10−12^

These characteristics make NBs very promising for use in a wide range of cutting-edge applications.^13−15^ NBs are most frequently used in environmental remediation, industrial wastewater treatment, pesticide removal, field irrigation, solid surface cleaning, and many other applications.^16−18^ A recent review describes the mechanisms of interaction between NBs and particles toward increasing particle separation efficiency.^12^ NBs are employed as the contrast agent for ultrasound sonography imaging (USG),^19^ and future medical applications may include drug administration and tumor killing.^20^ Plants and aquatic life also thrive miraculously when irrigated with NB containing water.^21^ In addition, because of these differential elements, nanobubbles offer intriguing opportunities to learn more about gas–liquid interfaces and develop novel transformative applications.^22^

**Figure 1:**
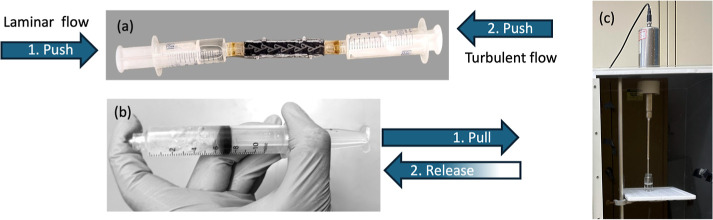
Laboratory-scale batch methods of nanobubble production. (a) 3-D printed Tesla valve connected to two 10 mL Luerlock syringes. Laminar flow is obtained by depressing the plunger on the left (forward direction), while turbulent flow is created by depressing the plunger on the right (reverse direction). (b) Pressure cycling using a 10 mL Luer Lock Syringe. (c) Sonication of a carbonated water sample with a Misonix Ultrasonic Liquid Processor.

There are numerous methods reported for producing NBs. A few notable examples include the hydrodynamic cavitation methods such as the spiral-liquid-flow generator,^23−25^ the Venturi-tube/nozzle-based generator,^26^ ejector-type generators,^27^ nanoporous membranes,^13^ depressurization methods,^28,29^ periodic pressure change,^30^ and ultrasonication methods.^31^ NBs can also be generated using alternating magnetic field, which has relatively low power requirements compared to other methods.^32^ Despite all the available methods, several disadvantages have been noted^33^ including that these methods are often highly dependent on process parameters or characteristics such as flow regime, heat generation, and phase pressures. Some methods also require high energy inputs and high costs of instrumentation. Thus, there is still a need to improve upon or develop new NB generation methods.^33^ Here, we are introducing a novel, cost-effective, sustainable, and durable method for easy generation of NBs using the Tesla valve. The Tesla valve is a valve without moving parts, also known as a valvular conduit.^34^ Nikola Tesla invented this technology and received a US patent in 1920. Promising applications of the Tesla valve have been demonstrated in microfluididics and pulse jet engines.^35−37^ Since it has no moving parts, it provides durability, scalability, and ease of fabrication.^38,39^ An example of a NB generator using the Tesla valve is shown in Figure 1a. Using the Tesla valve, it is possible to induce turbulent flow of fluids at much lower Reynolds numbers.^40^ The Reynolds number (*Re*), a dimensionless quantity that gauges the relationship between inertial and viscous forces, aids in the prediction of fluid flow patterns under various conditions. Laminar flow (*Re* < 4000) predominates in flows at low Reynolds numbers, whereas turbulent flow (*Re* > 4000) occurs at high Reynolds numbers. In a Tesla valve, the flow is different in the forward and reverse directions. The Tesla valve can produce early turbulence at Reynolds numbers as low as 400 in the reverse flow because of its complex geometry. While flow is laminar in the forward direction, reversing the flow results in a significant flow barrier due to the induced turbulence. Studies shows that when the length to depth ratio (*L*/*D*) of the valve is less than 40, early turbulence can be achieved in the reverse direction at low *Re*.^40^

Here, we compare the generation of NBs using flow regime switching in the Tesla valve with two other laboratory-scale NB batch generation methodologies, ultrasonication,^31^ and pressure cycling.^41^ Our comparison includes bubble size distributions, concentration, and zeta potential. Size distributions were determined by dynamic light scaterring and nanoparticle tracking analysis, which also provide bubble numbers.

## 2 Materials and Methods

### 2.1 Chemicals and Materials

Carbonated 3% ethanol solution was obtained using a SodaStream soda water maker (Mount Laurel, New Jersey, USA). Nitrogenated water was produced by bubbling pure N_2_ (Airgas, Gwinnett, Georgia) into to Milli-Q water (18.5 MΩ·cm) (Thermo Scientific, Langenselbold, Germany) for 5 min at a tank pressure of 50 psi and volumetric flow rate of 5.8 L/min. A 99.5% pure ethyl alcohol was obtained from Sigma-Aldrich (St. Louis, Missouri). Siraya Tech Blu resin from Siraya Tech (San Gabriel, California) was used for 3D printing. Polypropylene syringes (10 mL) were used (Norm-Ject Luer lock sterile syringes, Germany).

### 2.2 Sample Preparation

Carbonated and nitrogenated aqueous 3% ethanol solutions were used as samples. All samples were prepared at room temperature. The same N_2_ or carbonated sample was split between the three compared methods for subsequent NB generation.

### 2.3 NB Generation Methods

#### 2.3.1 Tesla Valve (Flow Regime Cycling)

A Tesla valve was 3D-printed with a length of 84 mm and channel dimesions of 1 by 4 mm (width x depth) to provide an *L*/*D* ratio = 21. When the length to depth ratio (*L*/*D*) of the valve is less than 40, early turbulence can be achieved in the reverse direction at low *Re*.^40^ The valve has a unique geometry of linked and looped lanes known as filaments, which provides its potential application. The Tesla valve used in this study had 10 filaments. The Tesla valve was printed using a Phrozen Sonic Mini 8K LCD Resin 3D printer using Siraya Tech Blu 3D printer resin. The print file was based on a plan published by Yunhao Bao et al.^42^ As shown in Figure 1a, 10 mL polypropylene syringes were connected to both ends of the valve body. The syringe on the left was filled with 5 mL of the carbonated or nitrogenated solvent. Upon depressing the syringe, solvent flows through the Tesla valve into the syringe on the right under laminar flow conditions. Next, the syringe on the right was depressed to push the solvent through the valve into the left syringe under turbulent flow. Whether turbulent or laminar flow occurs depends on the nonsymmetrical flow paths in the opposing directions. One cycle comprises a forward and backward directional flow, as shown in Figure 1a. The syringe stopper was depressed at a constant nominal rate of 2.5 mL/s. This resulted in linear flow rates in the forward and reverse directions of 0.625 m/s. The estimated Reynolds number for flow in the 1 × 4 mm channel with a characteristic linear dimension of 1.6 mm was *Re* = 1800, indicating laminar flow in an unimpeded flow channel. However, the flow is likely turbulent in the impeded reverse flow direction, as previously reported.^40^

#### 2.3.2 Pressure Cycling Method

A 10 mL polypropylene syringe^43^ was filled with 5 mL of the carbonated solvent sample, and the syringe tip was sealed with a Luer lock cap after any trapped air was released. The process is illustrated in Figure 1b. Once the syringe plunger was quickly pulled, the water was depressurized. This was followed by an instantaneous release of the plunger, which travels at a relatively high speed due to the pressure differential.^29,41^ The effective operation of this technique depends on maintaining an airtight syringe and generating the appropriate level of vacuum pressure; any leakage can compromise the internal pressure and, consequently, the efficiency of the NB formation process.

#### 2.3.3 The Sonication Method

Ultrasonication is a commonly employed method for generating nanobubbles, utilizing high-frequency sound waves to induce cavitation and form bubbles at the nanoscale.^44^ A 15 mL aliquot of carbonated or nitrogen-sparged solvent was sonicated in a 30 mL glass vial using a Misonix Ultrasonic Liquid Processor XL-2020 probe (Misonix Farmingdale, NY) for 5 min at 10 kHz.^5,45^ The simple experimental setup is shown in Figure 1c.

### 2.4 Characterization

#### 2.4.1 Bubble Size Distribution

The size distributions of nanoscale bubbles after the treatment by each of the NB generation methods were measured using dynamic light scattering (DLS) (Dynapro Titan, Wyatt Technology Corporation, Santa Barbara, CA). The DLS instrument was operated at 20% power of a 220W laser beam at 589 nm using a cuvette with a 1 cm light transmission channel.^46^

#### 2.4.2 Determination of the Concentration of Bubbles Formed

The number of bubbles formed after the treatment with each optimized NB generation method was determined by the PMX-230-Z-TWIN-488/640 Laser Zeta View Nanoparticle Tracking Analysis system (NTA) (Particle Metrix, Ammersse, Germany). Measurement settings were as follows: camera sensitivity (82), shutter (110), and cell temperature (24.09 °C). The video analysis parameters were maximum area (1000), minimum area (10), and minimum brightness (18).

#### 2.4.3 Solution pH Measurements

pH measurements were conducted using a Seven Easy S20 pH meter (Mettler Toledo, Switzerland). A four-point calibration was achieved using buffer with pH = 1.48, 4, 7, and 10.

Reported average values were for 6 measurements of independently carbonated and nanobubble generated solutions.

## 3 Result and Discussion

### 3.1 Optimization of Tesla Valve Cycle Number

The repeated cycling of the flow direction in the Tesla valve, with the subsequent cycling of flow regimes between laminar and turbulent flows, leads to the efficient formation of NBs, as shown in Figure S1.

The cycle number was optimized using cycle numbers of 3, 6, 10, 12, 16, and 20 of a 5 mL carbonated solution containing 3% ethanol in water. After the solution was repeatedly passed through the Tesla valve, we determined the radius of bubbles formed using DLS. At the optimized value of 12 cycles, around 50% of the generated bubbles had radii binned at 86 and 114 nm. Cycle numbers up to 10 also generated NBs but with a wider distribution that included microbubbles.

### 3.2 Optimization of Cycle Number for the Pressure Cycling Method

The repeated pull–release cycles of a syringe plunger leads to the efficient formation of NBs, as shown in Figure S2.

The cycle number was optimized using cycle numbers of 10, 20, 30, 40, 50, 60, 70, 80, 90, and 100 of a 5 mL carbonated solution containing 3% ethanol in water. The solution was repeatedly depressurized by pulling the plunger, and the radius of bubbles formed was deterimined using DLS. At the optimized value of 60 cycles, the generated bubbles had radii between 117 and 300 nm.

### 3.3 Optimization of Sonication Time for the Ultrasonication Method

The sample was subjected to different sonication times of 5, 10, 15, 20, and 25 min. The size distribution of produced nanobubbles by ultrasonication is shown in Figure S3. The optimum was found at 5 min where radii ranged between 60 and 555 nm.

### 3.4 Size Comparison between Nanobubbles Formed by Tesla Valve, Pressure Cycling, and Sonication

**Figure 2:**
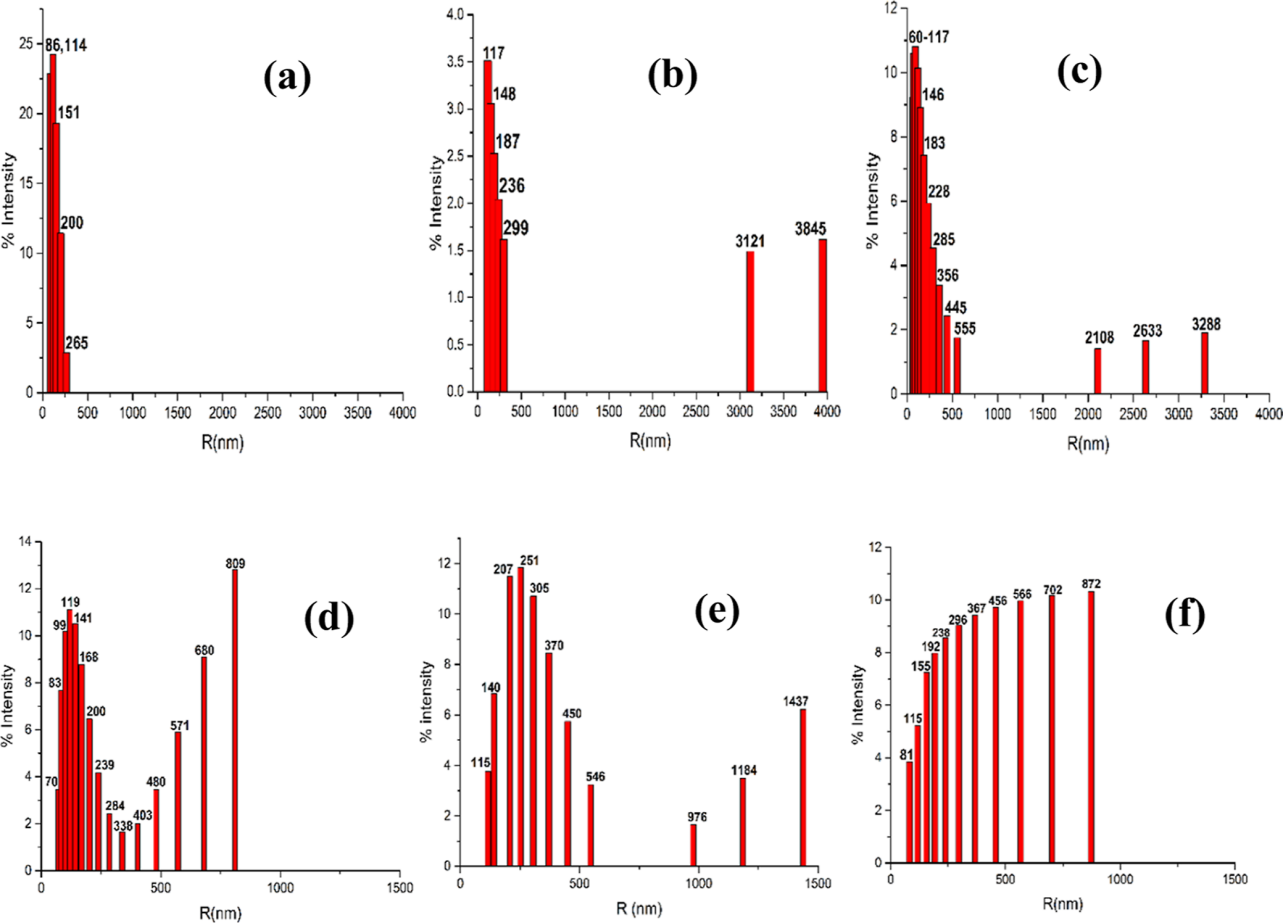
Radius distributions of bubbles formed under optimized conditions in 3% ethanol from CO_2_ by (a) Tesla valve, (b) pressure cycling, and (c) sonication and from N_2_ by (d) Tesla valve, (e) pressure cycling, and (f) sonication.

The bubbles formed at optimized conditions for each method were measured by DLS and compared in Figure 2.

The radii of CO_2_ bubbles measured from the three different methods indicate the Tesla valve’s ability to produce small NBs with tighter radii distribution and higher percentage formation ranging from 86 to 265 nm as compared to those created by pressure cycling (117–299 nm) and sonication methods (60–555 nm). In addition, under these conditions, no microbubbles were created by the Tesla valve.

The Tesla valve flow regime cycling method produced bubbles with a weighted average comparable to sonication with radii of 128 and 120 nm, respectively. Pressure cycling produced larger weighted average bubbles sizes of 168 nm.

When NBs were prepared from N_2_, the Tesla valve flow switching method produced bubbles with radii 70–571 nm as compared to those created by pressure cycling (115–546 nm) and sonication methods (81–566 nm). While the spread in bubble sizes are similar, the Tesla valve produced bubbles with a higher bubble fraction in the smaller bubble region. As previously published, smaller bubbles are obtained from CO_2_ than with N_2_.^47^

Bubble sizes were also determined by NTA, one of the most popular approaches to characterize NBs,^12^ as shown in Figure S4. For CO_2_ NBs produced by the three methods under optimized conditions, median diameters of 74 nm, 86 nm, and 68 nm were obtained for the Tesla valve, pressure cycling, and sonication, respectively. Weighted average sizes are provided in Table 1. DLS measures the fluctuations of the bulk scattering intensity, which is heavily influenced by larger particles in the sample, leading to an apparent larger average size than NTA. NTA tracks individual particles and their Brownian motion and is not biased toward larger particles.^48−51^

**Table 1:** Diameter, Concentration, Zeta Potential, and pH of CO_2_ NBs under Optimized Conditions of Each Method Measured by NTA^a^

method	weighted average size		concentration		zeta potential (mV)	pH
	diameter (nm)	% relative Std. Dev.	bubbles/mL	% relative Std. Dev		
Tesla valve	110	8.3	3.79 × 10^8^	7.4	–33.30	4.34 ± 0.031
pressure cycling	143	3.6	2.61 × 10^9^	19.2	–21.27	4.28 ± 0.030
sonication	99	11.0	2.99 × 10^8^	14.0	–36.17	4.39 ± 0.029

a Reported averages and RSDs are based on three independently produced NB samples for each method.

### 3.5 Bubble Number and Zeta Potential Comparison between Tesla Valve, Pressure Cycling, and Sonication

The average size of bubbles, concentrations, and zeta potential are shown in Table 1. Size distributions by NTA are shown in Figure S4. The weighted average sizes follow the same sequence as DLS. Measurement with both methods show pressure cycling produces the largest weighted average droplet sizes. Those produced by the Tesla valve and sonication were smaller.

The pressure cycling method produces a greater number of bubbles, which is followed by Tesla valve and sonication methods.

Zeta potential results indicate that the generation method also influences the surface charge of the bubbles. Sonication and the Tesla valve method have higher zeta potentials compared to pressure cycling. This correlated with the change in pH measured for carbonated solutions before and after the creation of NBs. A starting pH of 4.23 increases to 4.39 and 4.34 for sonication and Tesla valve methods, respectively, but only to 4.28 for pressure cycling.

Elsewhere, the nanobubbles produced with the three methods were compared for their use in electrospray ionization mass spectrometry. NBs significantly enhance the signal intensity of small molecules, with those generated using the Tesla valve method showing greater signal intensity increases compared to those generated using the other methods.^52^ Additionally, NBs produced by the Tesla valve and pressure cycling methods unfold proteins during electrospray ionization, whereas NBs generated by sonication do not.^53^ These differential chemical effects observed during electrospray ionization do not correlate with the physical measurements typically used to characterize NBs, as shown in Table 1, and will be the subject of future investigations.

## 4 Conclusions

The generation of NBs by flow regime switching using a Tesla valve appears to be equivalent to or better than comparative standard laboratory-scale batch methods of NB preparation. Under optimized conditions, the Tesla valve produced small bubbles and zeta potentials comparable to those by sonication. In addition, this technique has the advantage of requiring fewer cycles compared to the pressure cycling method, not requiring a high-wattage, high-frequency alternating current power supply as for sonication, and is thus cost-effective compared to other methods, promising the ability for large-scale implementation. In this work, only one Tesla valve design with a length to depth ratio of 21 was used. Future optimization of the Tesla valve, specifically for NB production, will likely lead to further improvements in performance.

## Supporting Information Available

Optimization of the NB generation methods (Tesla valve, pressure cycling method, and ultrasonication method) using DLS and the size distribution of CO_2_ bubbles under the optimized condition of each method using NTA (PDF)
