# Supplementary material for: Nanobubble Formation by Flow Regime Switching Using a Tesla Valve
Source: ACS Omega. 2025 Apr 14;10(16):16230–5. doi: 10.1021/acsomega.4c10246 (PMC12044576; doi:10.1021/acsomega.4c10246)
Supplement: Supplementary file 1 — ao4c10246_si_001.pdf [file ao4c10246_si_001.pdf]

Supplemental Information

Nanobubble formation by flow regime switching using a Tesla Valve

*George Joseph, Bincy Binny, Andre R Venter**

Department of Chemistry, Western Michigan University, Kalamazoo, Michigan, US, 49008-5413

andre.venter@wmich.edu

Table of Contents

Figure S1. Optimization of flow cycles of the Tesla Valve for the creation of nanobubbles.

Figure S2. Optimization of cycles of the pressure cycling method for the creation of nanobubbles.

Figure S3. Time optimization of ultrasonication method.

Figure S4. Size distribution of CO_2 bubbles under the optimised condition by (a) Tesla valve, (b) Pressure cycling, (c) Sonication methods using NTA.

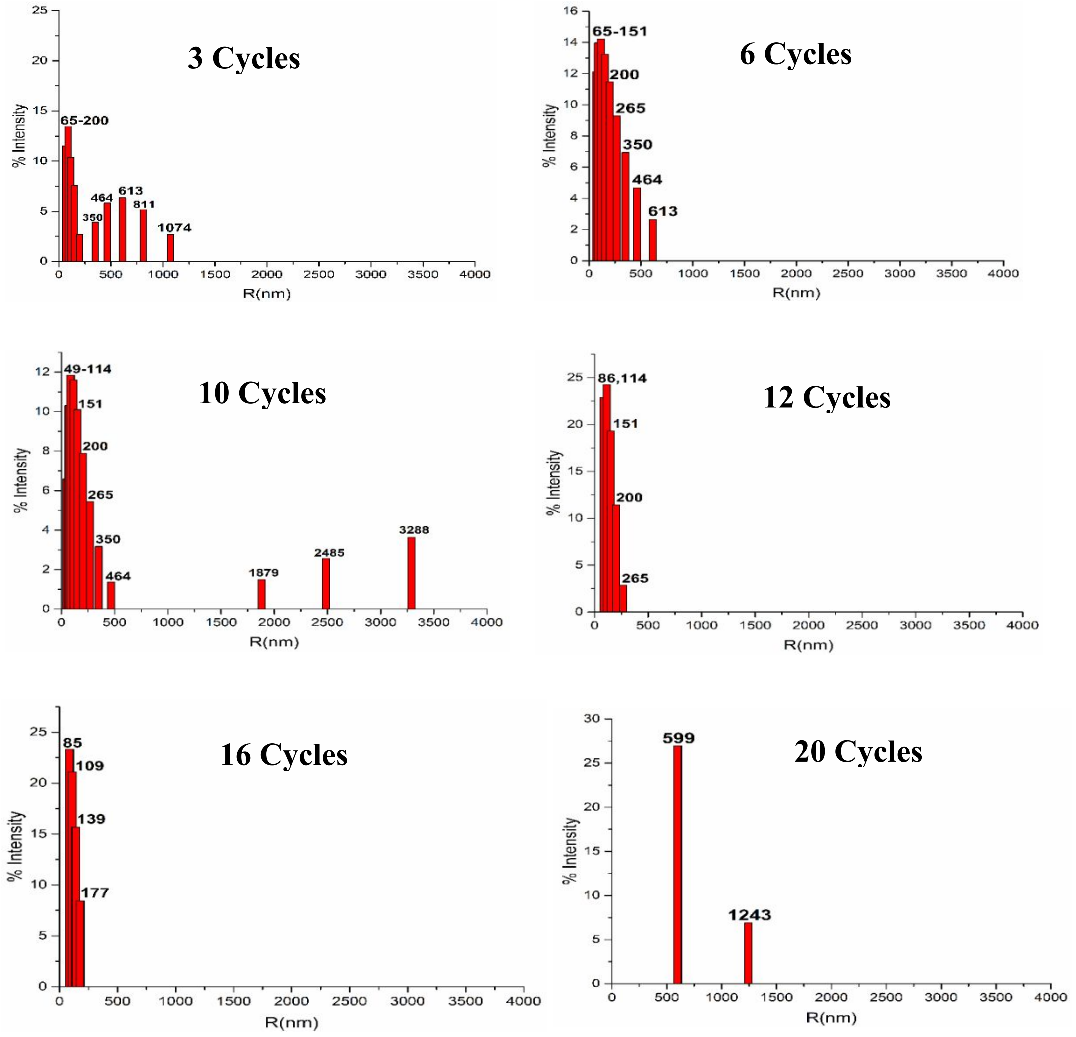

Figure S1. Optimization of flow cycles of the Tesla Valve for the creation of nanobubbles.

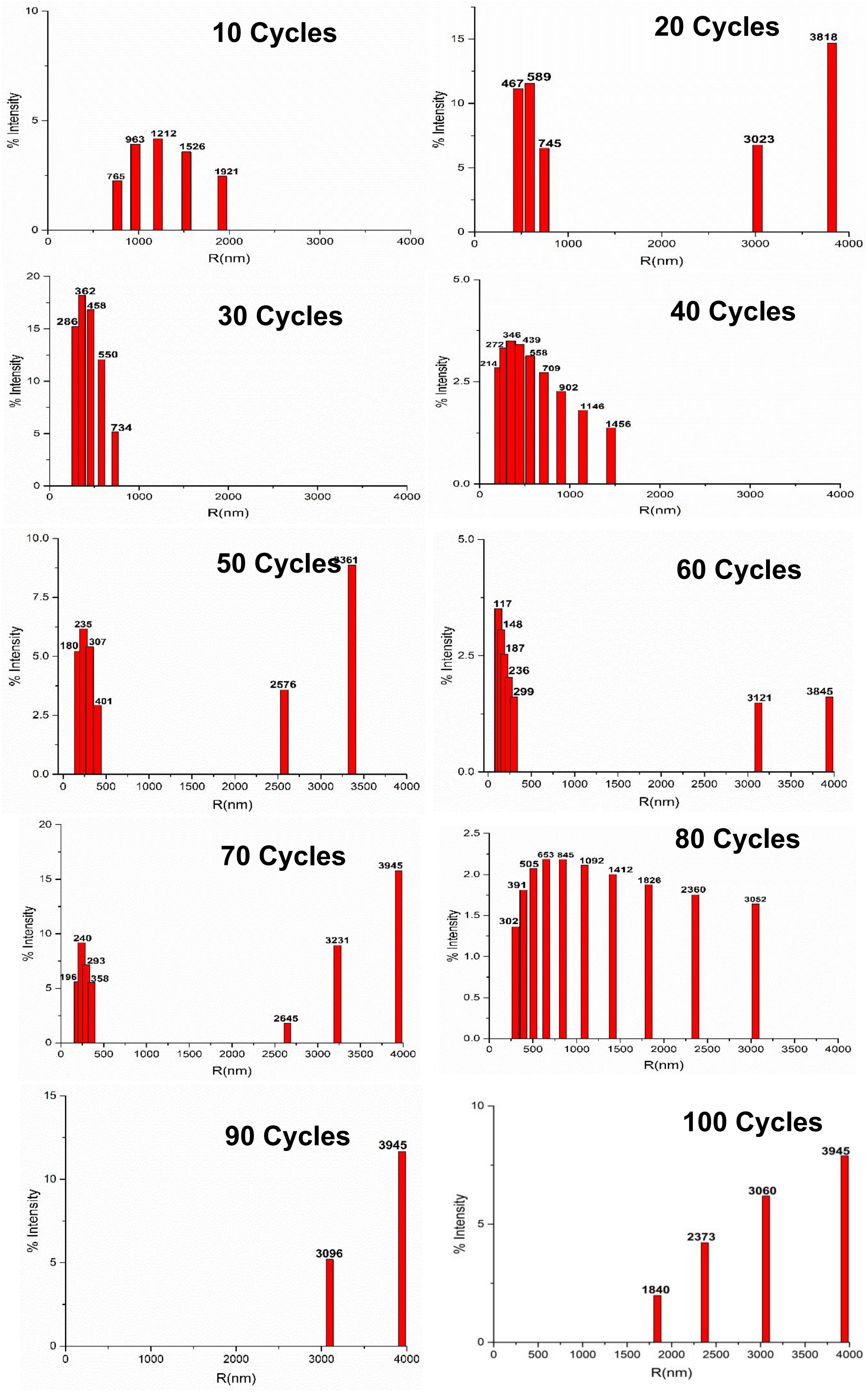

Figure S2. Optimization of cycles of the pressure cycling method for the creation of nanobubbles.

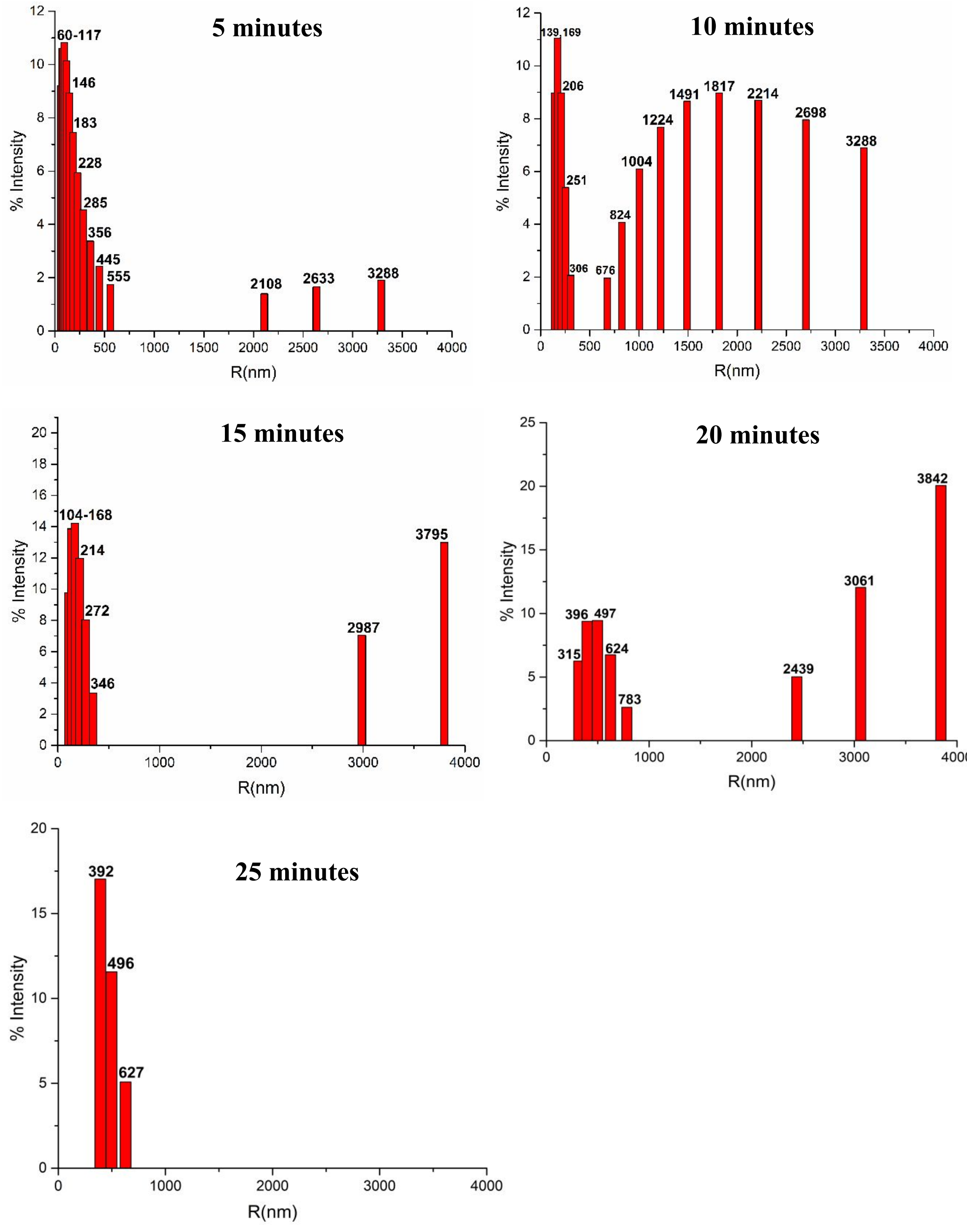

Figure S3. Time optimization of ultrasonication method**.**

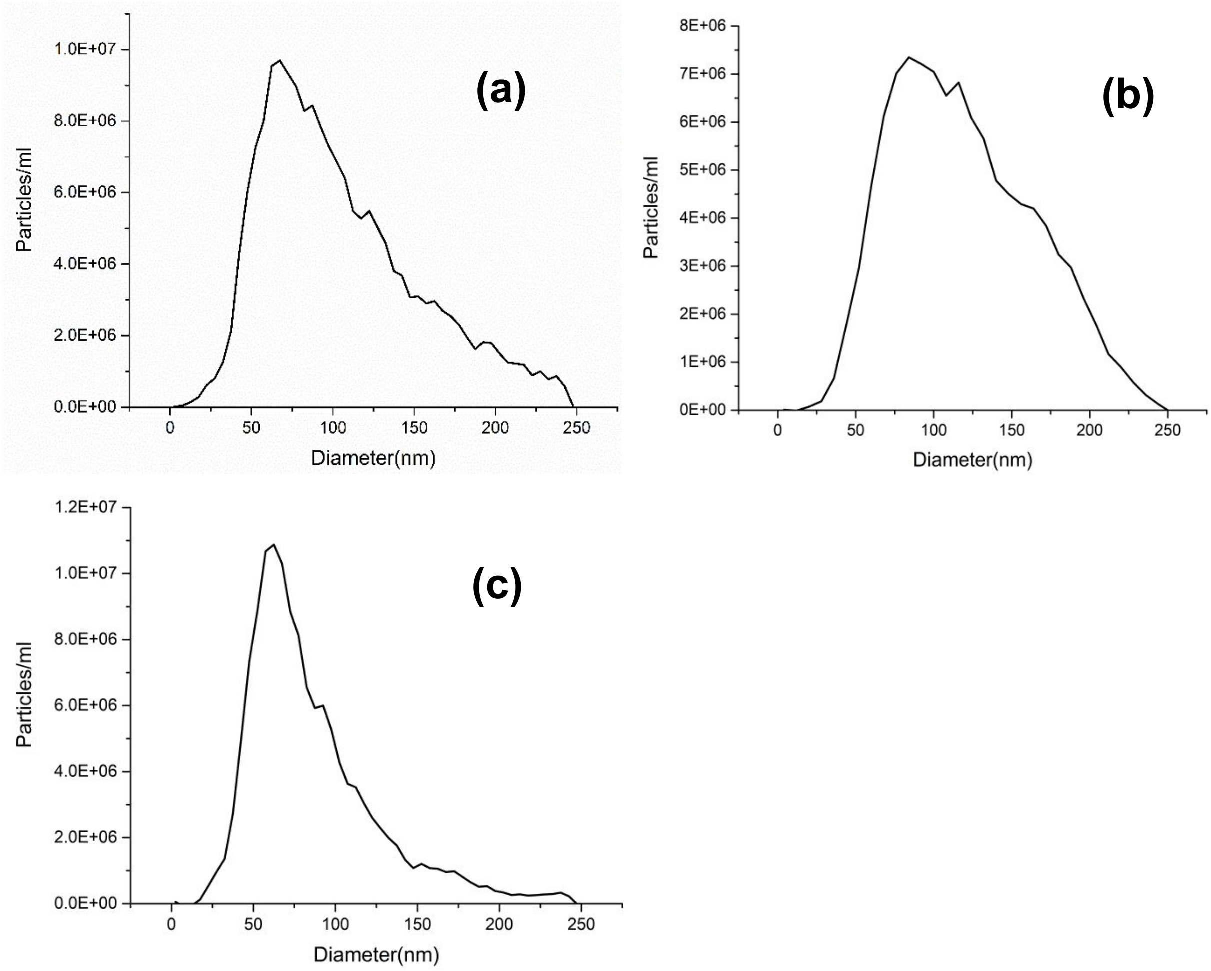

Figure S4. Size distribution by NTA of CO_2 bubbles under the optimised condition by (a) Tesla valve, (b) Pressure cycling, (c) Sonication methods.
